# Tissue-specific regulation of *Igf2r/Airn* imprinting during gastrulation

**DOI:** 10.1186/s13072-015-0003-y

**Published:** 2015-03-14

**Authors:** Chelsea Marcho, Ariana Bevilacqua, Kimberly D Tremblay, Jesse Mager

**Affiliations:** Department of Veterinary and Animal Sciences, University of Massachusetts at Amherst, ISB 427M, 661 N. Pleasant Street, Amherst, MA 01003 USA

## Abstract

**Background:**

Appropriate epigenetic regulation of gene expression during lineage allocation and tissue differentiation is required for normal development. One example is genomic imprinting, which is defined as parent-of-origin mono-allelic gene expression. Imprinting is established largely due to epigenetic differences arriving in the zygote from sperm and egg haploid genomes. In the mouse, there are approximately 150 known imprinted genes, many of which occur in imprinted gene clusters that are regulated together. One imprinted cluster includes the maternally expressed *Igf2r*, *Slc22a2,* and *Slc22a3* genes and the paternally expressed long non-coding RNA (lncRNA) *Airn.* Although it is known that *Igf2r* and *Airn* are reciprocally imprinted, the timing of imprinted expression and accompanying epigenetic changes have not been well characterized *in vivo*.

**Results:**

Here we show lineage- and temporal-specific regulation of DNA methylation and histone modifications at the *Igf2r/Airn* locus correlating with differential establishment of imprinted expression during gastrulation. Our results show that *Igf2r* is expressed from both alleles in the E6.5 epiblast. After gastrulation commences, the locus becomes imprinted in the embryonic lineage with the lncRNA *Airn* expressed from the paternal allele and *Igf2r* restricted to maternal allele expression. We document differentially enriched allele-specific histone modifications in extraembryonic and embryonic tissues. We also document for the first time allele-specific spreading of DNA methylation during gastrulation concurrent with establishment of imprinted expression of *Igf2r*. Importantly, we show that imprinted expression does not change in the extraembryonic lineage even though maternal DMR2 methylation spreading does occur, suggesting distinct mechanisms at play in embryonic and extraembryonic lineages.

**Conclusions:**

These results indicate that similar to preimplantation, gastrulation represents a window of dynamic lineage-specific epigenetic regulation *in vivo*.

**Electronic supplementary material:**

The online version of this article (doi:10.1186/s13072-015-0003-y) contains supplementary material, which is available to authorized users.

## Background

Genomic imprinting is an epigenetic phenomenon that results in mono-allelic gene expression in a parent-of-origin manner. Imprinted expression has been identified at approximately 150 mouse genes, which often occurs in clusters containing multiple imprinted transcripts [1,2]. Expression of imprinted genes is thought to be established in *cis* by allele-specific DNA methylation established at imprinting control regions (ICRs) in the gametes, thus arriving in the zygote as maternal and paternal specific information. A regulatory theme has emerged at many imprinted clusters in which a single long non-coding RNA (lncRNA) is thought to repressively regulate genes in *cis* through direct transcriptional blocking and/or recruitment of repressive chromatin remodeling complexes such as G9a and PRC2, resulting in differential allele-specific histone modifications [3,4].

One cluster on mouse chromosome 17 includes the maternally expressed *Igf2r*, *Slc22a2,* and *Slc22a3* genes and the paternally expressed lncRNA *Airn* [5], and several non-imprinted genes (*Slc22a1, Mas,* and *Plg).* The *Airn* promoter lies in the second intron of *Igf2r,* and *Airn* transcription occurs from the opposite strand overlapping *Igf2r* exons 1 and 2 [5-7]. Paternal *Airn* expression may participate in imprinting of the maternally expressed genes by blocking access of the transcriptional machinery to the *Igf2r* start site [8], and transcription of *Airn* has been shown to be required for silencing of *Igf2r* [8,9]. Paternal allele silencing of the other imprinted genes in the cluster only occurs in extraembryonic lineages and may be a result of *Airn* recruitment of repressive complexes such as G9a to their promoters [4]. Biallelic expression of *Igf2r* is observed in ES cells and only becomes imprinted upon differentiation *in vitro* [4]. Although the expression of *Igf2r* and *Airn* has been documented in preimplantation and late stage embryos [10-12], lineage-specific expression dynamics have not been observed during gastrulation. Recent studies have focused on mechanisms in ES cell models [4,8,13], but the precise timing and mechanisms responsible for imprinting at *Igf2r/Airn in vivo* remain unknown. Here we characterize tissue-specific dynamics of expression and epigenetic modifications that occur at *Igf2r*/*Airn* during normal gastrulation. We show that significant epigenetic regulation occurs at imprinted loci during epiblast differentiation *in vivo*.

## Results and discussion

### Imprinted expression of *Igf2r* and *Airn* during gastrulation

**Figure 1:**
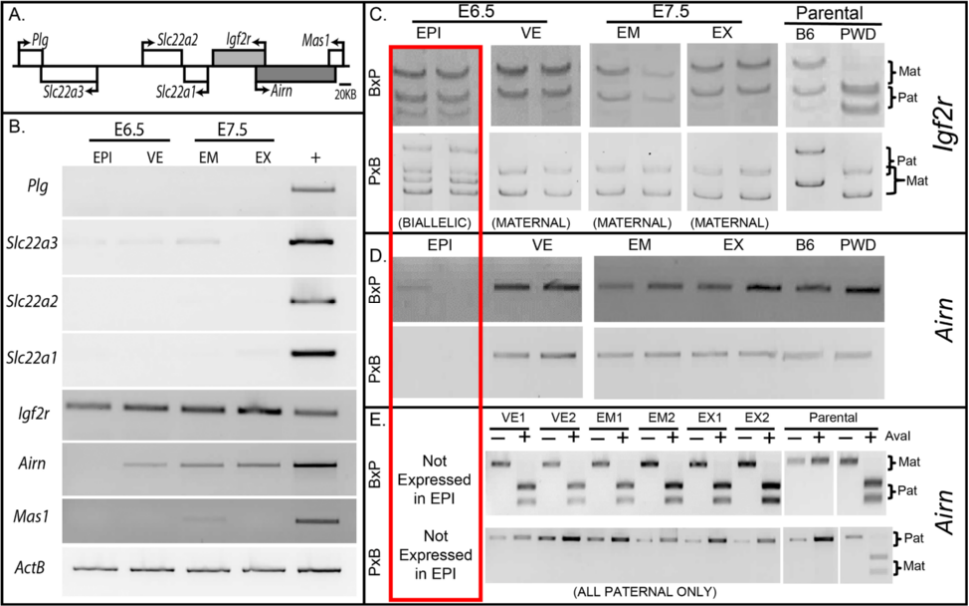
**Expression analysis. (A)** Schematic of gene locations at the mouse *Igf2r/Airn* locus: transcription start sites (bent arrows), *Igf2r* (light grey), and *Airn* (dark grey). **(B)** RT-PCR analysis of genes in the cluster shows expression of *Igf2r* at E6.5 and E7.5, as well as *Airn* expression in the E6.5 VE and E7.5 EM and EX. The other genes in the cluster are not expressed at appreciable levels during gastrulation. **(C)** SSCP analysis of *Igf2r* expression shows biallelic expression in the E6.5 EPI, while the paternal allele is silent (imprinted) in all other tissues and stages examined. **(D)** RT-PCR demonstrates that *Airn* is not expressed in epiblast but is paternally expressed **(E)** in all other samples. Two embryos (one per lane) shown for each tissue/stage for each assay. EPI, epiblast; VE, visceral endoderm; EM, embryonic portion of E7.5 embryo; EX, extraembryonic portion of E7.5 embryo. Red box highlights the non-imprinted status of *Igf2r* and lack of *Airn* expression in the E6.5 EPI. B, B6 allele; P, PWD allele. +, pooled adult kidney, liver, brain, and heart cDNA. Parental tissue used in (C-E) is adult kidney.

The *Igf2r/Airn* imprinted cluster contains the maternally expressed *Igf2r, Slc22a2,* and *Slc22a3,* the paternally expressed *Airn,* and the non-imprinted *Plg, Slc22a1,* and *Mas1* genes (Figure 1A). We determined the expression of the genes within the cluster in embryonic and extraembryonic tissues from C57BL/6JxPWD/PhJ-F1 embryos at embryonic days E6.5 and E7.5 by RT-PCR (Figure 1B). Of the genes in the cluster, *Igf2r* is expressed in both the epiblast (EPI) and visceral endoderm (VE) at E6.5 and the embryonic (EM) and extraembryonic (EX) tissues of E7.5 embryos (Figure 1B). *Airn* is expressed in the VE at E6.5 and in both tissues at E7.5. However, no *Airn* was detected in the epiblast at E6.5 (Figure 1B).

To further understand the imprinted expression of *Igf2r* and *Airn* during gastrulation, we carried out allele-specific expression analysis of C57BL/6JxPWD/PhJ-F1 and C57BL/6J-Chr 17^PWD/Ph/ForeJ^xC57BL/6J-F1 embryos (hereafter referred to as B × P and P × B F1 embryos, respectively). Single-strand confirmation polymorphism (SSCP) revealed that *Igf2r* is expressed from both alleles in the EPI of E6.5 embryos (Figure 1C, red box). In E6.5 VE, *Igf2r* is maternally expressed and paternally imprinted (Figure 1C). At E7.5, *Igf2r* is imprinted in both tissues (Figure 1C). Our results show that in the multipotent epiblast, *Igf2r* is expressed from both alleles, but once embryonic cells have adopted defined lineages at E7.5, *Igf2r* expression becomes imprinted. This correlation suggests a relationship between relative differentiation state *in vivo* and imprinted expression at the locus - consistent with ES cell models.

Since *Airn* is thought to establish imprinting of *Igf2r* [8], we also examined allele-specific *Airn* expression. In E6.5 EPI, *Airn* is not expressed (Figure 1B,D, red box), corresponding with biallelic *Igf2r* expression (Figure 1C). In the VE at E6.5, where *Igf2r* is imprinted, we observe reciprocal imprinting (paternal expression) of *Airn* (Figure 1E). At E7.5, *Igf2r* and *Airn* are imprinted in both embryonic and extraembryonic tissue (Figure 1E). This change in imprinted expression between EPI and EM also occurs in the reciprocal cross (P × B, Figure 1C,D,E), ruling out background-specific genetic differences. *Airn* has also been shown to regulate imprinting of *Slc22a2* and *Slc22a3* in extraembryonic lineages [5]; however, we could not detect these transcripts at appreciable levels during gastrulation (Figure 1B). The change in *Igf2r* and *Airn* expression indicate a lineage- and stage-specific establishment of imprinted expression during normal development. We therefore examined allele-specific epigenetic modifications at the locus.

### DNA methylation spreads at DMR 2

**Figure 2:**
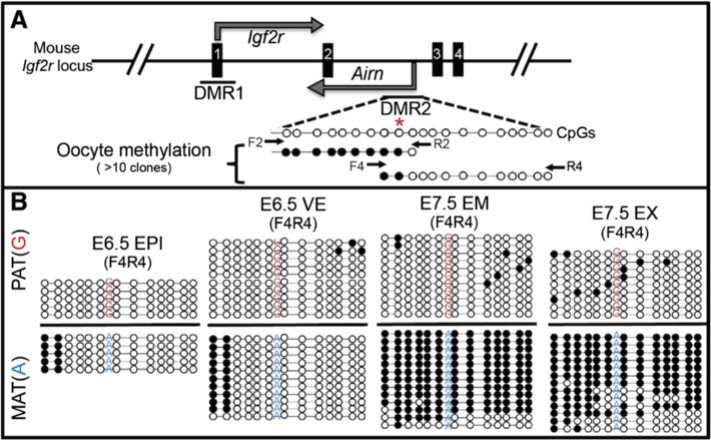
**DNA methylation at DMR2. (A)** Schematic of the mouse *Igf2r/Airn* locus: transcription start sites (bent arrows), *Igf2r* exons (solid boxes), and location of DMR1 and DMR2. Two amplicons (F2R2 and F4R4) spanning 20 CpG dinucleotides were analyzed. Methylation in oocytes confirmed that DMR2 is an ICR and defined the methylation boundary (red asterisk in A). **(B)** ICR methylation is maintained in EPI and VE, but spreading of maternal DMR2 methylation occurs in EM and EX (compare EPI to EM, and VE to EX). Filled circles = methylated cytosine, Open = unmethylated cytosine. Asterisk denotes ICR border. Arrows indicate bisulfite-sequencing primer locations in **(A)**. Parental SNP indicated on each bisulfite strand. Pat, paternal; Mat, maternal.

Allele-specific DNA methylation at ICRs or differentially methylated regions (DMRs) are required for imprinted expression at many loci [14-16]. The *Igf2r/Airn* locus has two known differentially methylated regions (Figure 2A, [15]). Methylation at DMR1 has been shown to occur late in development in a tissue-specific manner after imprinting is established and is thought to be a consequence of *Airn* expression [17]. In all tissues that we examined during gastrulation, DNA methylation at DMR1 is not significantly different than methylation in the adult brain [12] (Additional file 1: Figure S1), suggesting that DMR1 DNA methylation does not regulate the silencing of the paternal *Igf2r* allele that we observe at E7.5.

DMR2 methylation has been shown to be present in oocytes [17], defining DMR2 as an ICR. Previous reports documented DMR2 by methylation-sensitive restriction enzymes, presenting the analysis of two specific CpG dinucleotides [13,17]. To gain a more comprehensive understanding of the methylation status, we designed two overlapping PCR amplicons for bisulfite sequencing at DMR2 (Figure 2A). With this approach, we fortuitously identified the precise 3′ boundary of ICR methylation present in oocytes [CpG at Chr17:12,742,488-12,742,489 (Figure 2A, red asterisk)]. In E6.5 EPI and VE, the precise ICR border was maintained on the maternal allele (Figure 2B). However, by E7.5 DNA methylation had spread in the 3′ direction in both embryonic and extraembryonic tissues (Figure 2B). These results indicate that although ICR methylation at DMR2 is established in the female germline [17], maternal allele-specific methylation increases/spreads in cells of all lineages coincident with the onset of gastrulation. It is particularly intriguing that the methylation spreading occurs in the extraembryonic tissue given that reciprocal imprinting of *Igf2r* and *Airn* is already established. It is also evident that the increase in DNA methylation is coincident with initiation of *Airn* expression in the epiblast, suggesting a tissue-specific mechanistic relationship. It may be of interest in the future to determine how far DNA methylation continues, if the spreading also occurs in the 5′ direction, and if the spreading is required for paternal silencing of *Igf2r* and activation of *Airn*.

### *Airn* is progressively expressed during development

**Figure 3:**
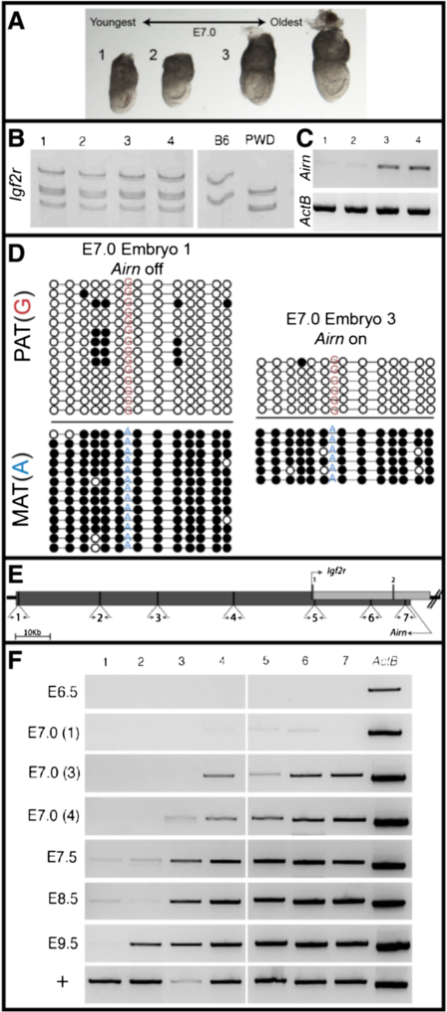
**Mid-gastrulation expression and DNA methylation. (A)** Four approximately E7.0 embryos from a single litter shown in age order - analyzed for *Igf2r* **(B)**, *Airn* and *ActB* **(C)** expression as well as DMR2 DNA methylation **(D)**. *Igf2r* is expressed from both alleles in all four embryos but *Airn* is detected only in the two older embryos (3 and 4). DNA methylation has already spread across DMR2 (D) independent of *Airn* expression. Filled circles = methylated cytosine, Open = unmethylated cytosine. **(E)** Schematic of *Airn* illustrating *Igf2r* start site and locations of primer pairs used in **(F)**. (F) RT-PCR expression analysis from approximately E7.0 embryos 1, 3, 4, as well as other stages and adult kidney RNA highlight progressive expression of *Airn* coordinated with development. Pat, paternal; Mat, maternal; +, kidney cDNA.

To more closely examine the timing of *Airn* expression, paternal *Igf2r* silencing, and the spread of DNA methylation at DMR2, we carefully assessed litters of late-streak stage embryos at approximately E7.0 to establish relative developmental age within each litter (Figure 3A). The spreading of maternal methylation at DMR2 has already occurred in all embryos examined at approximately E7.0 (Figure 3A shows four embryos from the same litter). Surprisingly, *Igf2r* is expressed from both alleles in these same embryos (Figure 3B). However, *Airn* is only expressed in slightly older embryos (3 and 4 (Figure 3C)). Hypermethylation of maternal DMR2 (Figure 3D) clearly precedes paternal *Airn* expression in embryos 1 and 2. Hypermethlyation of maternal DMR2 also precedes silencing of paternal *Igf2r* in embryonic lineages (embryos 3 and 4). Importantly, the epiblast of embryos 3 and 4 express both alleles of *Igf2r* and paternal *Airn*, suggesting that transcription from both loci can occur on opposite strands of the same paternal chromosome. Analysis with single cell-resolution will be important to support this finding.

Since the *Airn* transcript is 108 kb, we designed amplicons along its length to assess if the entire lncRNA is detectable in embryonic lineages at various developmental stages (Figure 3E). Qualitative RT-PCR indicates that *Airn* transcripts increase in length with developmental progress (Figure 3F). At E7.0, only the 5′-most amplicons are detected while the 3′-most amplicons are also detected in older embryos (Figure 3F). By E9.5, all but the very 3’-most amplicon is detected, and the entire lncRNA is detectable in adult tissue (Figure 3F, +). Although qualitative, these results also suggest that total levels of *Airn* transcripts increase as development proceeds (Figure 3F, compare to *ActB* control).

These findings raise the possibility that maternal DNA methylation spreading is required to inhibit maternal *Airn* transcription. This could explain activation of only the paternal unmethylated allele in the embryo. Furthermore, the difference in *Igf2r* and *Airn* expression between EPI (biallelic *Igf2r* and no *Airn*) and VE (reciprocal imprinting) indicate a distinct mechanism during epiblast differentiation that activates *Airn* (since *Airn* is already expressed in VE but the methylation dynamics are the same in EPI-EM and VE-EX). Alternatively, there may be regulation on the paternal allele that initially inhibits *Airn* transcription in epiblast. Either scenario indicates that neither DNA methylation nor *Airn* expression is responsible for silencing paternal *Igf2r* in the epiblast. Taken together, the observation that DNA methylation dynamics are the same in embryonic and extraembryonic tissues but that allelic expression patterns are different, require an embryonic lineage-specific mechanism responsible for establishment of imprinted expression.

### Ctcf binding at DMR2

**Figure 4:**
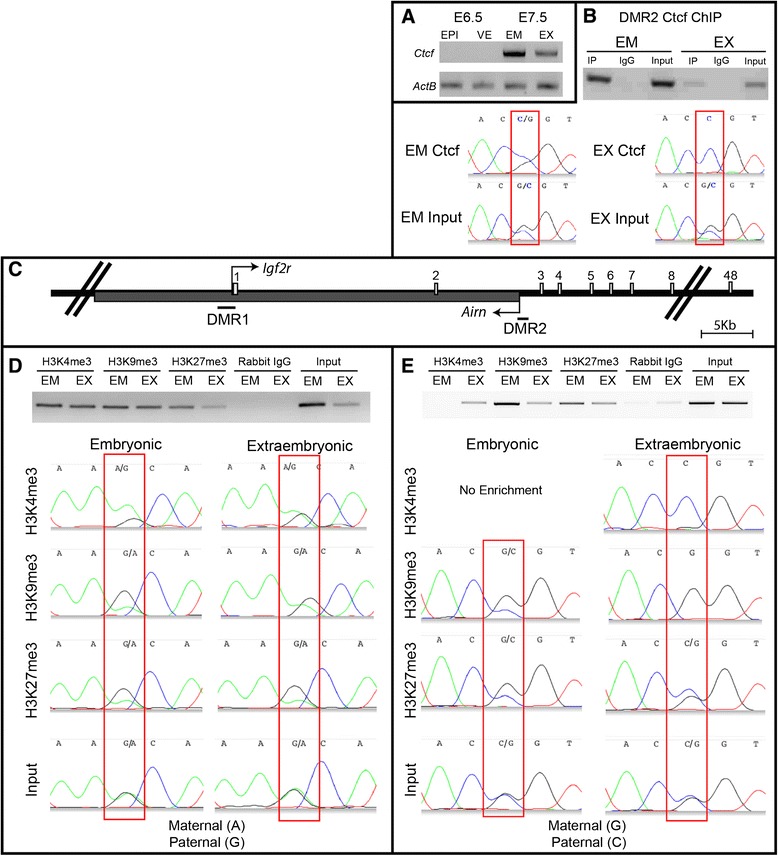
**Chromatin modification at DMR2. (A)** *Ctcf* RT-PCR indicates no/trace expression of *Ctcf* in both EPI and VE at E6.5. *Ctcf* is expressed in both lineages at E7.5. **(B)** Ctcf ChIP at DMR2 with E8.5 chromatin shows binding in both embryonic and extraembryonic tissues. Sequencing of ChIP-PCR products indicates allele-specific Ctcf enrichment of the paternal allele in the extraembryonic tissue, but both alleles are bound in embryonic tissue (although biased toward the paternal allele). **(C)** Illustration of DMR1 and DMR2 in relation to the start sites of *Igf2r* and *Airn*. **(D-E)** H3K4me3, H3K9me3, and H3K27me3 ChIP-PCR of DMR1 and DMR2 on E8.5 chromatin. Sequencing of ChIP-PCR products shows allele-specific enrichment in embryonic tissues at DMR1 and extraembryonic tissue at DMR2. The position of the polymorphism is boxed in red on each electropherogram. DMR1 (A, maternal; G, paternal). DMR2 (G, maternal B6; C, paternal PWD).

The methylation-sensitive insulator Ctcf participates in chromatin looping at imprinted loci [18-20]. At *Igf2/H19*, Ctcf binds to the unmethylated maternal ICR preventing *Igf2-*enhancer interactions [19,21]. We therefore examined Ctcf expression and binding to DMR2 to determine if it may be involved in regulation of *Igf2r/Airn.* At E6.5, very little *Ctcf* transcript is detectable in EPI or VE (Figure 4A), but expression is evident at E7.5 in both lineages. Consistent with the absence of transcripts, immunofluorescence showed no/trace nuclear signal at E6.5, while robust nuclear Ctcf is observed at E7.5 (Additional file 2: Figure S2). We confirmed earlier reports of *Ctcf* expression in blastocysts (both mRNA and protein, Additional file 2: Figure S2), indicating that the locus undergoes dramatic transcriptional regulation during normal development. This dramatic change in *Ctcf* expression and localization was unexpected and may reflect the important role that the protein plays in maintaining epigenetic regulatory domains. Perhaps Ctcf is required during preimplantation to establish and/or maintain chromatin dynamics established during the first cell lineage decisions (ICM/TE) but is dispensable until the next major lineage decisions are made during gastrulation. This possibility supports the idea that genome-wide epigenetic alterations are required during gastrulation lineage decisions, similar to preimplantation. In the future, conditional deletion strategies may make it feasible to functionally test the requirement of Ctcf in specific tissues during gastrulation.

Ctcf chromatin immunoprecipitation (ChIP) indicates Ctcf binds to DMR2 in both embryonic and extraembryonic E8.5 tissues (Figure 4B). Sequencing of ChIP-PCR products clearly shows allele-specific binding of Ctcf at DMR2 to the unmethylated paternal allele in extraembryonic tissues (Figure 4B). Surprisingly, both alleles are bound by Ctcf in embryonic lineage - although there is a detectable shift toward the paternal allele (compare input and ChIP, Figure 4B). Together, the lack of *Ctcf* in the epiblast at E6.5 and the biallelic binding of Ctcf at E8.5 suggest that while Ctcf may play a role in maintaining imprinted expression at later stages, it is not involved in the initiation of paternal *Igf2r* silencing or *Airn* activation in the embryonic lineage during gastrulation.

### Differential histone enrichment at DMR2

Allele-specific histone modifications (HMODs) have been shown to correlate with *Igf2r* imprinting in the central nervous system [12,22]. We therefore performed ChIP to examine enrichment of H3K4me3, H3K9me3, and H3K27me3 at DMR1 and DMR2 (Figure 4C). In the embryonic tissue at DMR1, we observe maternal allele-specific enrichment of H3K4me3, as well as paternally biased H3K9me3 and H3K27me3. In the extraembryonic tissues, there is no allele-specific enrichment of H3K4me3 and a weak paternal bias of H3K9me3 and H3K27me3 (Figure 4D).

Similar to binding of Ctcf, we observe allele-specific enrichment of HMODs at DMR2 in extraembryonic tissues but not in chromatin derived from the embryo (Figure 4E). In extraembryonic tissues, the active H3K4me3 mark is greatly enriched on the paternal allele (which expresses *Airn*), and H3K9me3 is enriched on the maternal allele (where *Airn* is silent, Figure 4E). Surprisingly, PRC2-mediated H3K27me3 which has been shown to be required for imprinting at other loci [23] is not enriched on the silent *Igf2r* allele, suggesting that PRC2 does not participate in regulation of the *Airn* locus in extraembryonic cells. In the embryonic tissue however, we find maternal bias of repressive H3K9me3 and H3K27me3 (although not as highly enriched as extraembryonic cells).

Together these data indicate distinct lineage- and allele-specific enrichment of HMODs occur at DMR1 and DMR2. Strikingly, there is limited allele-specific enrichment in the extraembryonic tissue at DMR1 at E8.5, even though *Igf2r* is imprinted at least 2 days prior. This indicates differential methylation (Additional file 1: Figure S1), and HMODs at DMR1 (Figure 4D) may play a secondary role in the imprinting of *Igf2r*.

Dramatic allele-specific binding/enrichment of Ctcf, H3K4me3, and H3K9me3 is present at DMR2 in extraembryonic tissues suggesting that these chromatin modifications are established at an earlier stage. Although biased, DMR2 allele-specific chromatin modifications are not fully established in embryonic lineages by E8.5. While it is possible that multiple cell types of the E8.5 embryo contain distinct allele-specific enrichment, it is more likely that the allele-specific modifications are not yet fully established - particularly since imprinted expression of *Igf2r* and *Airn* is initiated only 24 h prior at E7.5.

## Conclusions

**Figure 5:**
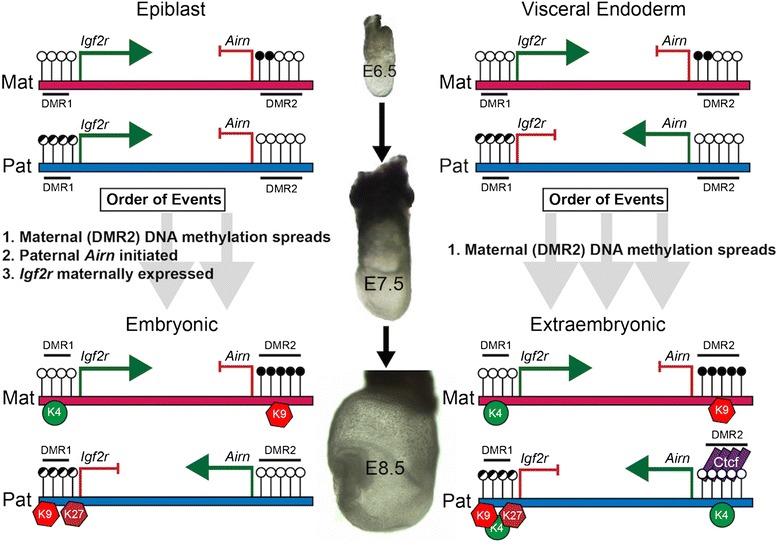
**Summary schematic depiction showing dynamic regulation of** ***Igf2r*** **and** ***Airn*** **in the embryonic lineage.** In the EPI, *Igf2r* is biallelic and *Airn* is not expressed. During gastrulation, maternal methylation at DMR2 spreads, followed by initiation of paternal *Airn* expression and then silencing of paternal *Igf2r*. In the extraembryonic lineage (right side), reciprocal imprinting of *Igf2r* and *Airn* is already established in VE at E6.5. DMR2 methylation spreads but does not correlate with changes in allelic expression in extraembryonic lineage. Paternal specific binding of Ctcf occurs at DMR2 in the extraembyronic lineage, but not in the embryo. Additionally, lineage- and allele-specific histone modifications are present at DMR1 and DMR2 suggesting an epiblast-specific mechanism required to establish imprinted expression.

Our results indicate lineage-specific regulation of *Igf2r/Airn* imprinted expression during gastrulation. We identify the precise ICR boundary as well as spreading of DNA methylation at *Igf2r* DMR2 during gastrulation (summarized in Figure 5). At E6.5, both EPI and VE lineages maintain maternal ICR methylation. However, the epiblast expresses biallelic *Igf2r* and no *Airn.* In contrast, both genes are imprinted in visceral endoderm of the same embryos. Therefore, there must exist mechanistic distinctions that result in imprinted expression in VE but not in EPI at E6.5 - possibly lineage-specific expression of chromatin binding/modifying genes established during preimplantation inner cell mass/trophectoderm differentiation. Our data also show locus-specific methylation spreading occurs during gastrulation in both lineages. While spreading of ICR methylation is known to occur during preimplantation, it has not been previously shown during gastrulation at imprinted loci, indicating that DNMTs are targeted to the locus specifically during these stages. The lineage differences in imprinted expression documented herein are remarkably similar to patterns of X-inactivation. Imprinted X-inactivation is established in extraembryonic cells during preimplantation, while stochastic X-inactivation occurs in the embryo only after gastrulation commences. Furthermore, non-coding RNAs help induce silencing in *cis* of the inactive X chromosome (reviewed in [24]) suggesting that *Airn* may function in a similar fashion at the *Igf2r* locus. The results presented here support the possibility that regulation of *Igf2r/Airn* (and other imprinted loci) and X-inactivation may utilize a common mechanism. Identification of the lineage-specific machinery that enables epigenetic changes specifically in the embryo will lead to a more complete understanding of the events underlying normal gastrulation and epigenetic reprogramming.

## Methods

### Tissues

All procedures were approved by the University of Massachusetts Amherst Institutional Animal Care and Use Committee. Embryos were derived from C57BL/6J (JAX 000664) and PWD/PhJ (JAX004660). Reciprocal F1 embryos were derived from female C75BL6/J Chr17^PWD/PhJ/ForeJ^ (JAX 005267) and C57BL/6J (JAX 000664) males. Embryos were microdissected for DNA and mRNA extraction. MII oocytes were collected from superovulated B6D2F1 females to confirm ICR methylation.

### Imprinted expression analysis

Total RNA was isolated using the Roche High Pure RNA Isolation Kit (Roche 11828665001, Roche, Basel, Switzerland). cDNA synthesis was performed using Bio-Rad iScript cDNA synthesis kit (170-8891) (Bio-Rad Laboratories, Inc., Hercules, USA). Primers for allele-specific expression and full-length *Airn* RT-PCR are shown in Additional file 3: Table S1. *Airn* restriction fragment length polymorphism (RFLP) was performed with AvaI. SSCP was performed on *Igf2r* PCR products with MDE polyacrylamide gel electrophoresis (Lonza 50620, Lonza Group, Basel, Switzerland). PCR products were visualized by ethidium bromide illumination and imaging.

### Bisulfite sequencing

Bisulfite sequencing was performed as previously described [25] with the primers provided in Additional file 3: Table S1.

### Chromatin immunoprecipitation

E8.5 C75BL/6JxPWD/PhJ-F1 embryos were dissected, and embryonic and extraembryonic tissues were separated and immediately processed using instructions in either ChIP-IT High Sensitivity kit (Active Motif 53040, Active Motif, Carlsbad, USA) or Zymo-Spin ChIP kit (Zymo D5210, Zymo Research, Irvine, USA). Samples were kept on ice and either sonicated twice for 20 s with the Heat Systems Sonicator/Ultra Processor (output 3) or sonicated for 30 s on/20 s off for 3 min using a cup horn adaptor for the QSonica A500 (QSonica, Newtown, USA). After sonication, 1% of each sample was removed for input control. Immunoprecipitation was carried out using Active Motif Protein G agarose beads or magnetic Protein G Dynabeads (10003D, Life Technologies, Carlsbad, USA) and either anti-Ctcf (Santa Cruz sc-28198, Santa Cruz Biotechnology, Inc., Dallas, USA), anti-H3K4me3 (Abcam ab8580, Abcam, Cambridge, UK), anti-H3K9me3 (Abcam ab8898), or H3K27me3 (Millipore 07-449, Millipore, Billerica, USA) along with normal rabbit IgG. After antibody incubation, beads were washed and DNA was collected using manufacturer’s protocol. ChIP-PCR primers found in Additional file 3: Table S1 (Additional file 4: Supplemental Methods).

## Additional files

Additional file 1: Figure S1. DMR1 methylation. (A) Schematic of the mouse *Igf2r/Airn* locus: transcription start sites (bent arrows), *Igf2r* exons (boxes), and location for DMR1 and DMR2. One amplicon spanning 12 CpG dinucleotides were analyzed. (B) Methylation of adult F1 brain and liver. Note DMR1 methylation levels are variable in a tissue specific context. (C) Embryo DMR1 methylation at E6.5, E7.0, and E7.5. Levels of DMR1 methylation are comparable to the adult brain. Maternal = A, Paternal = G. Additional file 2: Figure S2. CTCF immunoflourescence. (A, A’) No/trace nuclear CTCF signal is detectable in EPI or VE in E6.5 embryos. Decidual cells show robust nuclear CTCF (FITC) serving as a positive control. (B, B’) Robust nuclear CTCF is observed at E7.5 in all cells. (C, C’) Nuclear staining is observed in the blastocyst at E3.5. CTCF shown in FITC/green, nuclear counterstain DAPI shown in blue. Scale = 100 μm. Additional file 3: Table S1. Primers for allele-specific expression and full-length *Airn* RT-PCR. Additional file 4:
**Supplemental Methods.**

## Abbreviations

ChIP: chromatin immunoprecipitation
DMR: differentially methylated region
DNMT: DNA methyltransferase
EM: embryonic
EPI: epiblast
ES cells: embryonic stem cells
EX: extraembryonic
H3K27me3: histone H3 lysine 27 tri-methylation
H3K4me3: histone H3 lysine 4 tri-methylation
H3K9me3: histone H3 lysine 9 tri-methylation
HMODs: histone modifications
ICM: inner cell mass
ICR: imprinting control region
lncRNA: long non-coding RNA
PRC2: Polycomb Repressive Complex 2
RFLP: restriction fragment length polymorphism
SSCP: single-strand conformation polymorphism
TE: trophectoderm
VE: visceral endoderm
Xi: X-inactivation
