# Supplementary figures and images for: Tissue-specific regulation of Igf2r/Airn imprinting during gastrulation
Source: Epigenetics Chromatin. 2015 Mar 14;8:10. doi: 10.1186/s13072-015-0003-y (PMC4410455; doi:10.1186/s13072-015-0003-y)

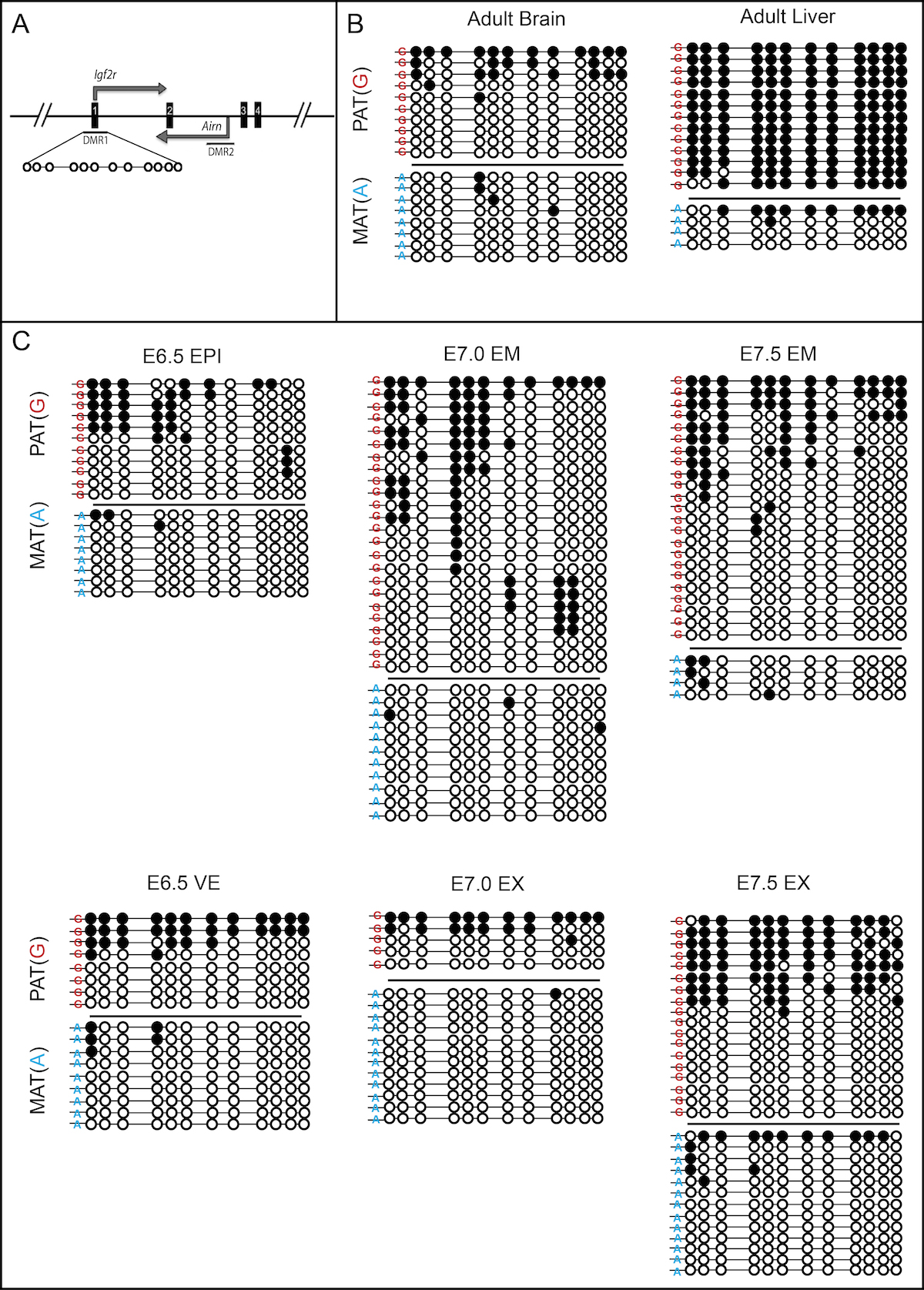

Supplement: Additional file 1: Figure S1. — DMR1 methylation. (A) Schematic of the mouse Igf2r/Airn locus: transcription start sites (bent arrows), Igf2r exons (boxes), and location for DMR1 and DMR2. One amplicon spanning 12 CpG dinucleotides were analyzed. (B) Methylation of adult F1 brain and liver. Note DMR1 methylation levels are variable in a tissue specific context. (C) Embryo DMR1 methylation at E6.5, E7.0, and E7.5. Levels of DMR1 methylation are comparable to the adult brain. Maternal = A, Paternal = G. [file 13072_2015_3_MOESM1_ESM.jpeg]

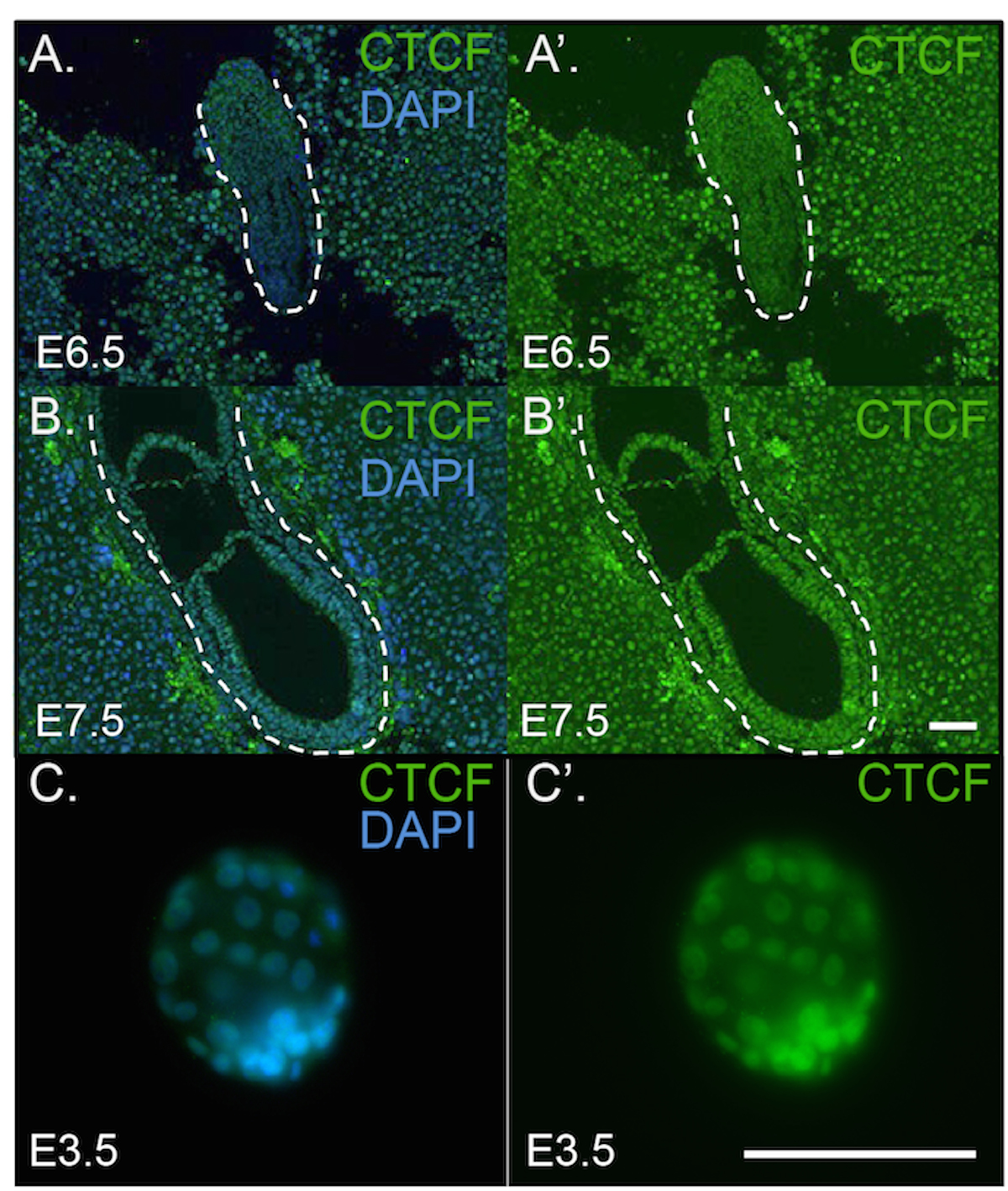

Supplement: Additional file 2: Figure S2. — CTCF immunoflourescence. (A, A’) No/trace nuclear CTCF signal is detectable in EPI or VE in E6.5 embryos. Decidual cells show robust nuclear CTCF (FITC) serving as a positive control. (B, B’) Robust nuclear CTCF is observed at E7.5 in all cells. (C, C’) Nuclear staining is observed in the blastocyst at E3.5. CTCF shown in FITC/green, nuclear counterstain DAPI shown in blue. Scale = 100 μm. [file 13072_2015_3_MOESM2_ESM.jpeg]
