# Supplementary material for: Tissue-specific regulation of Igf2r/Airn imprinting during gastrulation
Source: Epigenetics Chromatin. 2015 Mar 14;8:10. doi: 10.1186/s13072-015-0003-y (PMC4410455; doi:10.1186/s13072-015-0003-y)
Supplement: Additional file 3: Table S1. — Primers for allele-specific expression and full-length Airn RT-PCR. [file 13072_2015_3_MOESM3_ESM.docx]

| **Expression Analysis** |  |
| --- | --- |
| Plg_F1 | GATGAATTATTCCACAACC |
| Plg_R1 | TTTCTGGTGTCCTGTTGTGC |
| Slc22a3_F1 | AGAGAAAGCCTTGCAGATCC |
| Slc22a3_R1 | CAGTGACCAGACACGACACC |
| Slc22a2_F1 | CCAGTGCATGAGGTATGAGG |
| Slc22a2_R1 | GGCATAGTTGGGTGAAATCG |
| Slc22a1_F1 | TAACCTGGTGTGTGGAGACG |
| Slc22a1_R1 | CTGGTACAAGATGGCTGTCG |
| Igf2r_F (SSCP) | TTCGACCTATAAGAAGCCTT |
| Igf2r_R (SSCP) | GGGTACTTTGCTTTTGGGTA |
| Airn_F (RFLP) | GGGTGGAGCCTTATGATGAA |
| Airn_R (RFLP) | TGAAGCCTGGGTTTCATTTC |
| Mas1_F2 | TGGCAAAGGCAGGATCTATT |
| Mas1_R2 | TGTTACCAGACGGCAGGAAT |
| Airn_F1 | ATTCAGCCCTGCATACTTGG |
| Airn_R1 | GACGGGGTCTAGAATCACCA |
| Airn_F2 | TCCAGACCAGTCAAGGGTTT |
| Airn_R2 | ATAAGAAAGCAGGCCAAGCA |
| Airn_F3 | CAACAGGGACACCAGGAGTT |
| Airn_R3 | CCTGAAGTGGACACAGGTGA |
| Airn_F4 | CCCTCTACAAAGGCAGCAAG |
| Airn_R4 | CCCATCAGTCTTGGCAGAAT |
| Airn_F5 | GCACGAGCGCCAGGTACCTACTCGA |
| Airn_R5 | AGGTGGTGGTACACGCTTCT |
| Airn_F6 | GGAGTAGGGAGAACCTGCAA |
| Airn_R6 | AGAATGGGCTGTGTGTTTCC |
| Airn_F7 | CTAGGGTTCAGCGCAAATCT |
| Airn_R7 | CCCAAGATGTACCAGCCAGT |
| ActB_F | ACGGCCAGGTCATCACTATTG |
| ActB_R | ATGGATGCCACAGGATTCCAT |
| **Methylation Analysis** |  |
| DMR1F (Yamaski 2005) | GGGATTTTAGAAAGATTGATTTT |
| DMR1R (Yamaski 2005) | AAACCTAACAACCCCAAAATTACTCAC |
| BS_Igf2r_F4 | GGGTTGTGATTTTGGTTATGTTAAG |
| BS_Igf2r_R4 | ACAAAACCCTCTAAATCCTCCTATC |
| BS_Igf2r_F2 | TTAAGGGTGAAAAGTTGTATAAGGAG |
| BS_Igf2r_R2 | ACTTAACATAACCAAAATCACAACC |
| **Chromatin IP Analysis** |  |
| Chip1F-2 (Yamaski 2005) | GACTGACCTCTTAACCCTGC |
| Chip1R-2 (Yamaski 2005) | TTCAACCGAGACCAGTACG |
| ChIP_DMR2_F | TGATGAGAACTGGTGGGTTG |
| ChIP_DMR2_R | AGAGGGCTCTGCACTATCCA |
| ChIP_Slc22a3_F2 | CCTTCCTCAGTGCCTGGTC |
| ChIP_Slc22a3_R2 | GGAGCTGGAGGAATGTGATG |
