# Supplementary material for: Tissue-specific regulation of Igf2r/Airn imprinting during gastrulation
Source: Epigenetics Chromatin. 2015 Mar 14;8:10. doi: 10.1186/s13072-015-0003-y (PMC4410455; doi:10.1186/s13072-015-0003-y)
Supplement: Additional file 4: — Supplemental Methods. [file 13072_2015_3_MOESM4_ESM.docx]

**Supplemental Methods**

**Immunofluorescence:**

Decidua containing embryos were collected in cold PBS and were prepared for histology by fixation in 4% paraformaldyhe (PFA) for 1 hour at room temperature. Embryo/decidua were dehydrated through a series of ethanol washes for 15 minutes in 70%, 80%, 90% ethanol diluted in phosphate buffered saline (PBS), followed by three washes of 100% ethanol. Samples were infiltrated for 90 minutes in xylene at room temperature, then in 50% xylene/ 50% paraplast for 1 hour. They were then transferred to 100% paraplast and incubated overnight at 65°C. The samples were then embedded in plastic molds and cooled to room temperature. Using a microtome, 7 micrometer sections were cut and floated in water, collected on Superfrost Plus slides (Fisher 12-550-15) and dried overnight at 42°C. Sections were deparafinized with three 5-minutes washes in xylene and rehydrated with three 5-minute washed in 100% ethanol, followed by 2-minute washes in 90%, 80%, and 70% ethanol. Antigen retrieval was performed by boiling for 4 minutes in 0.01M Tris Base pH10. Once the slides cooled to room temperature, they were washed twice in phosphate buffered saline with 0.01% Tween-20 (PBT) for 2-minutes blocked with 0.5% milk in PBT for two hours at room temperature in a humidified chamber. Primary antibody was applied in 0.05% milk/PBT overnight at 4°C in a humid chamber. Three 15-minute PBT washes preceded a 1-hour secondary antibody treatment in 0.05% milk/PBT in a humid chamber at room temperature. Slides were washed in in PBS three times for 15 minutes. Nuclei were countered stained with Dapi (Roche or Molecular Probes) in PBS (1:10,000) for 2 minutes and then rinsed with PBS. Slides were sealed and coverslipped with Prolong Gold (Invitrogen).

Preimplantation immunofluorescence was performed on blastocysts collected from superovulated B6D2F1 females. Embryos were flushed from the uteri with PBS and fixed in 4% PFA/PBS for 30 minutes at room temperature. Samples were washed in PBS/0.1% Triton-X100 3 times, then permeabilized with PBS/0.5% Triton-X100 for 15 minutes at room temperature, and then washed in PBS/0.1% Triton-X100 3 times. Blocking was carried out for 1 hour at room temperature using PBS/10% FBS with 0.1% Triton-X100. Primary antibody was applied in PBS/10% FBS with 0.1% Triton-X100 at 4°C overnight. Samples were washed 3 times with PBS/0.1% Triton-X100 followed by 1-hour secondary antibody treatment in PBS/0.1% Triton-X100. After secondary antibody staining, samples were washed 3 times with PBS/0.1% Triton-X100 followed by counterstaining with Dapi and mounted on slides with 90% glycerin/PBS.

Primary antibody used was CTCF (Santa Cruz, sc28198) and secondary antibody used was Alexa Fluor-488 donkey anti-rabbit IgG (Invitrogen, A21206). Both antibodies were used at 1:500 dilution.
